# Supplementary material for: Deciphering the Hidden Ecology and Connectivity of Vibrio in the Oceans
Source: Nat Commun. 2026 Apr 1;17:4707. doi: 10.1038/s41467-026-71231-3 (PMC13212558; doi:10.1038/s41467-026-71231-3)
Supplement: Supplementary file 1 — Supplementary Information [file 41467_2026_71231_MOESM1_ESM.pdf]

Title

Deciphering the Hidden Ecology and Connectivity of *Vibrio* in the Oceans

Author list

Lapo Doni[1,2,3], Joaquin Trinanes[4], Emanuele Bosi[1], Luigi Vezzulli[1,2*+], Jaime Martinez-Urtaza[3,5*+]

Affiliations

1 Department of Earth, Environmental and Life Sciences (DISTAV), University of Genoa; 16132, Genoa, Italy.

2 NBFC, National Biodiversity Future Center; 90133, Palermo, Italy

3 Centre for Environment, Fisheries and Aquaculture Science (CEFAS); DT4 8UB, Weymouth, UK.

4 Department of Electronics and Computer Science, University of Santiago de Compostela; 15706, Santiago de Compostela, Spain.

5 Department of Genetics and Microbiology, Universitat Autònoma de Barcelona (UAB); 08193, Barcelona, Spain.

*Corresponding authors e-mails: luigi.vezzulli@unige.it, jaime.martinez.urtaza@uab.cat

+ Joint Supervision

Fig. S1.

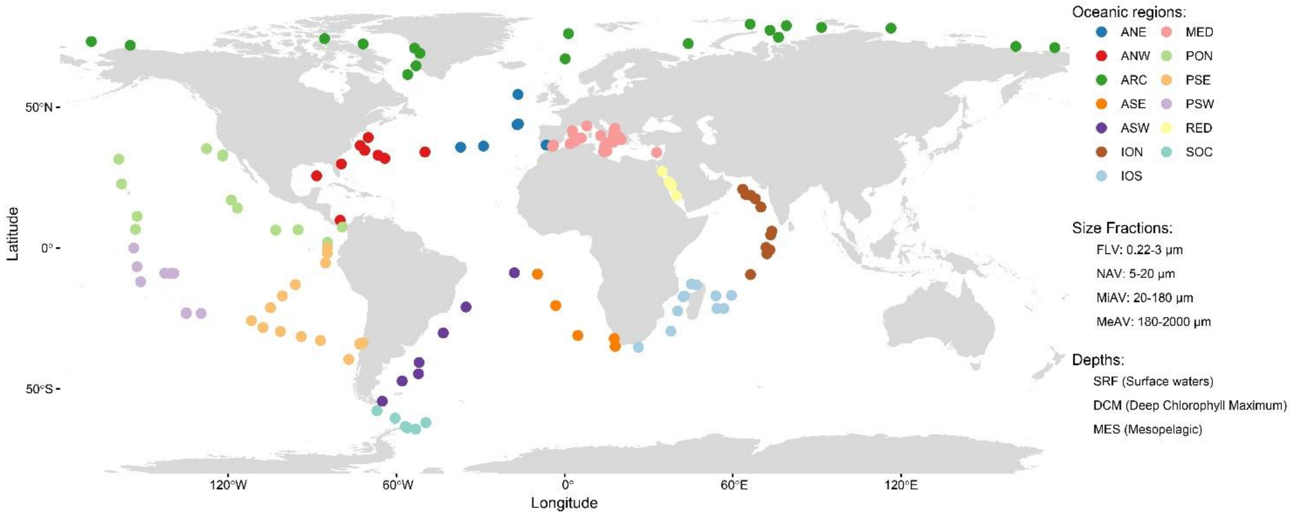

Global map of the 147 Tara Oceans sampling stations analyzed in this study. Oceanic regions are shown in different colors. Size fractions and sampling depths relevant to the stations are indicated.

Fig. S2.

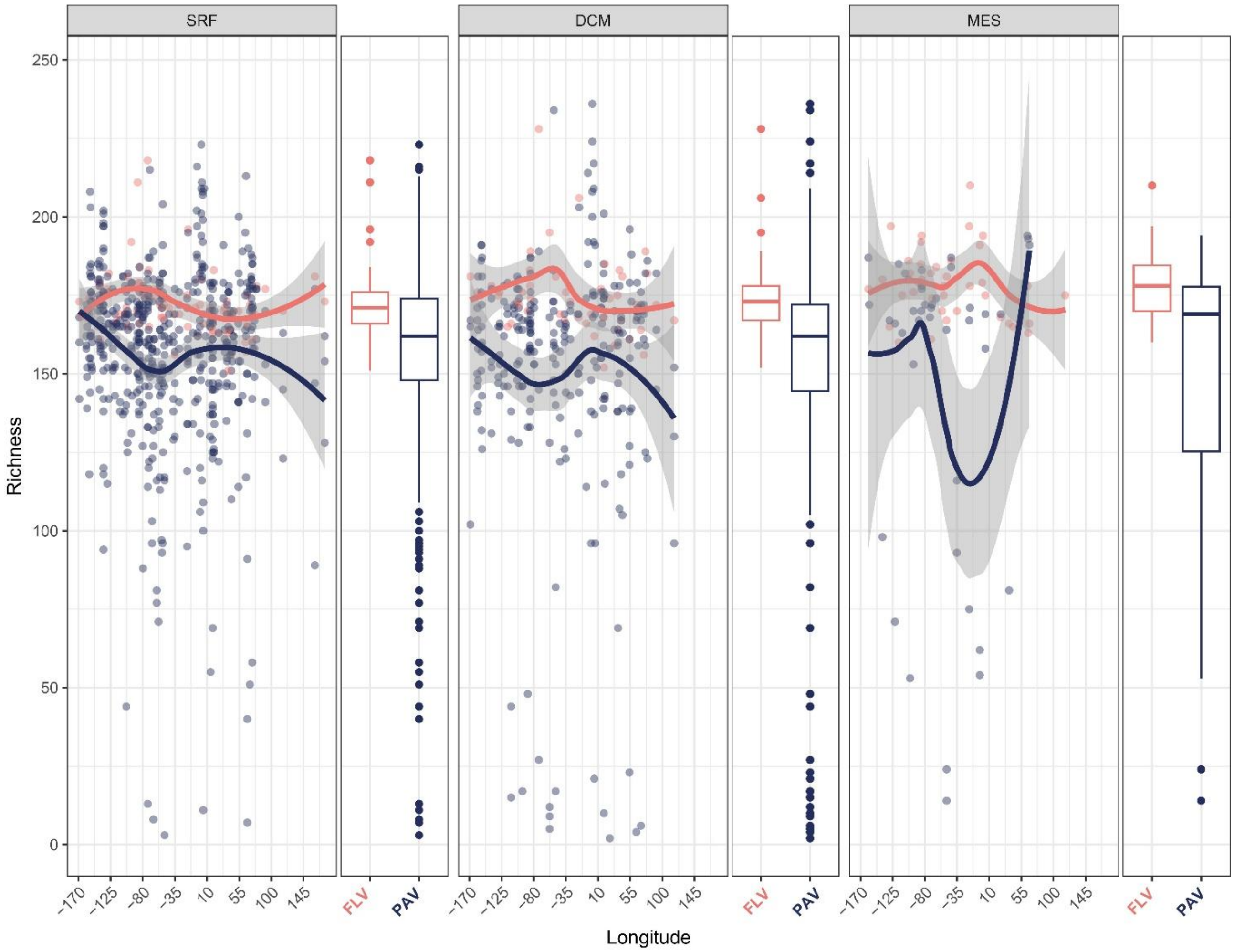

Vibrio Alpha Diversity and Longitude. *Vibrio* alpha diversity, measured as the species richness, was calculated only for samples in which *Vibrio* reads were classified at species level and plotted against the longitude of the stations for the different depths: SRF (Surface waters), DCM (Deep Chlorophyll Maximum) and MES (Mesopelagic). Colors represent FLV (free-living *Vibrio*) pink and PAV (plankton associated *Vibrio*) blue. The plot includes LOESS curves (shaded bands = 95% confidence interval), boxplots summarize richness values for each size fraction and depth: the center line represents the median, box bounds indicate the interquartile range (IQR) defined as the difference between the first quartile (Q1) and the third (Q3), whiskers extend to the minimum and maximum values within [Q1−1.5×IQR, Q3+1.5×IQR], horizontal lines represent median values. Sample sizes for SRF: n = 81 FLV and n = 550 PAV; for DCM: n = 49 FLV and n = 267 PAV; and for MES n = 39 FLV and n = 42 PAV.

Fig. S3.

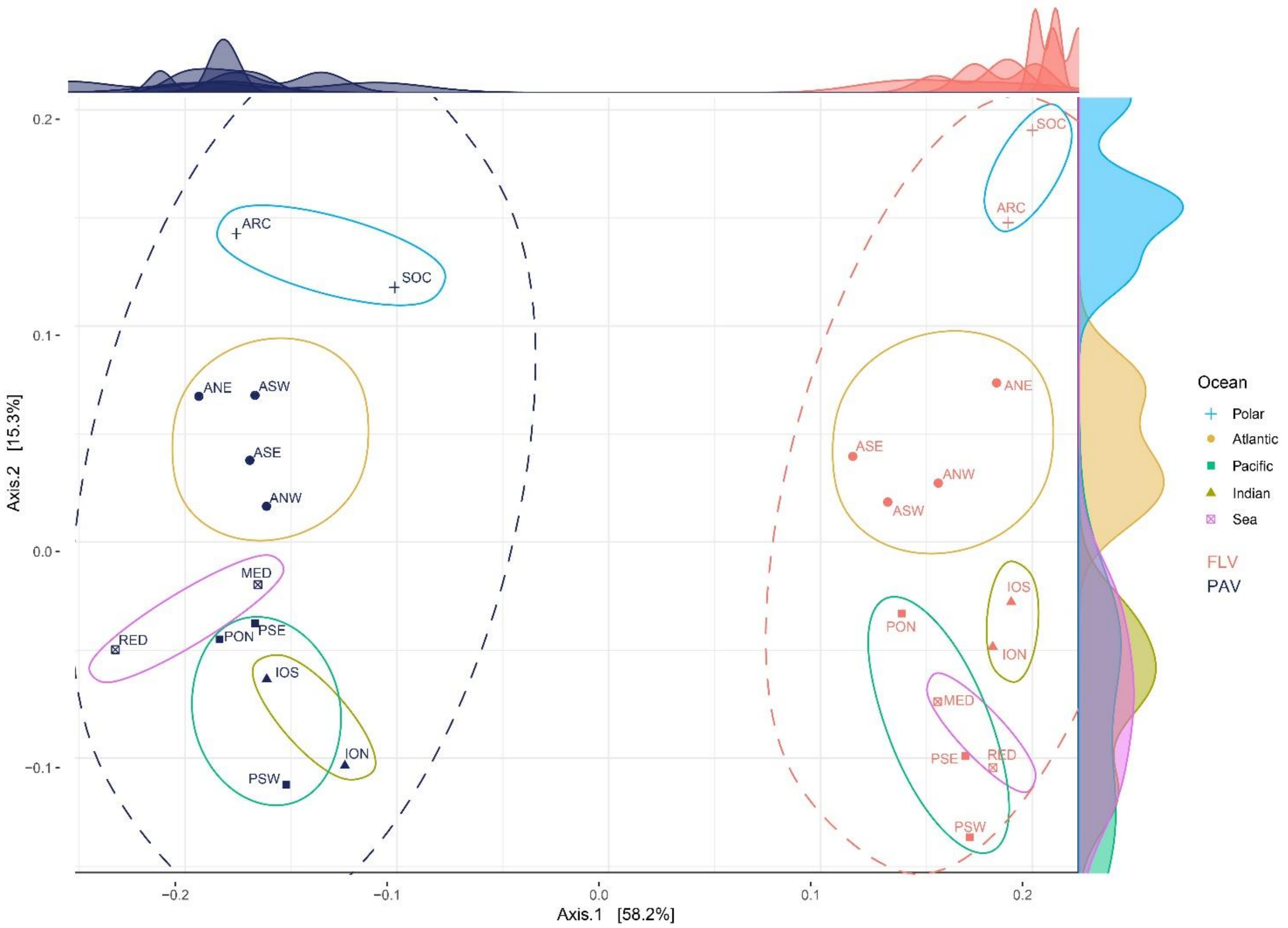

Beta diversity based on *Vibrio* taxonomy frequencies (t-PcoA). Dashed ellipses correspond to FLV (free-living *Vibrio*) (pink) and PAV (plankton associated *Vibrio*) (blue). Oceanic regions are indicated by the colors of the continuous ellipses and the shapes. The percentage of the variation explained by each axis is indicated in parentheses after the axis label.

Fig. S4.

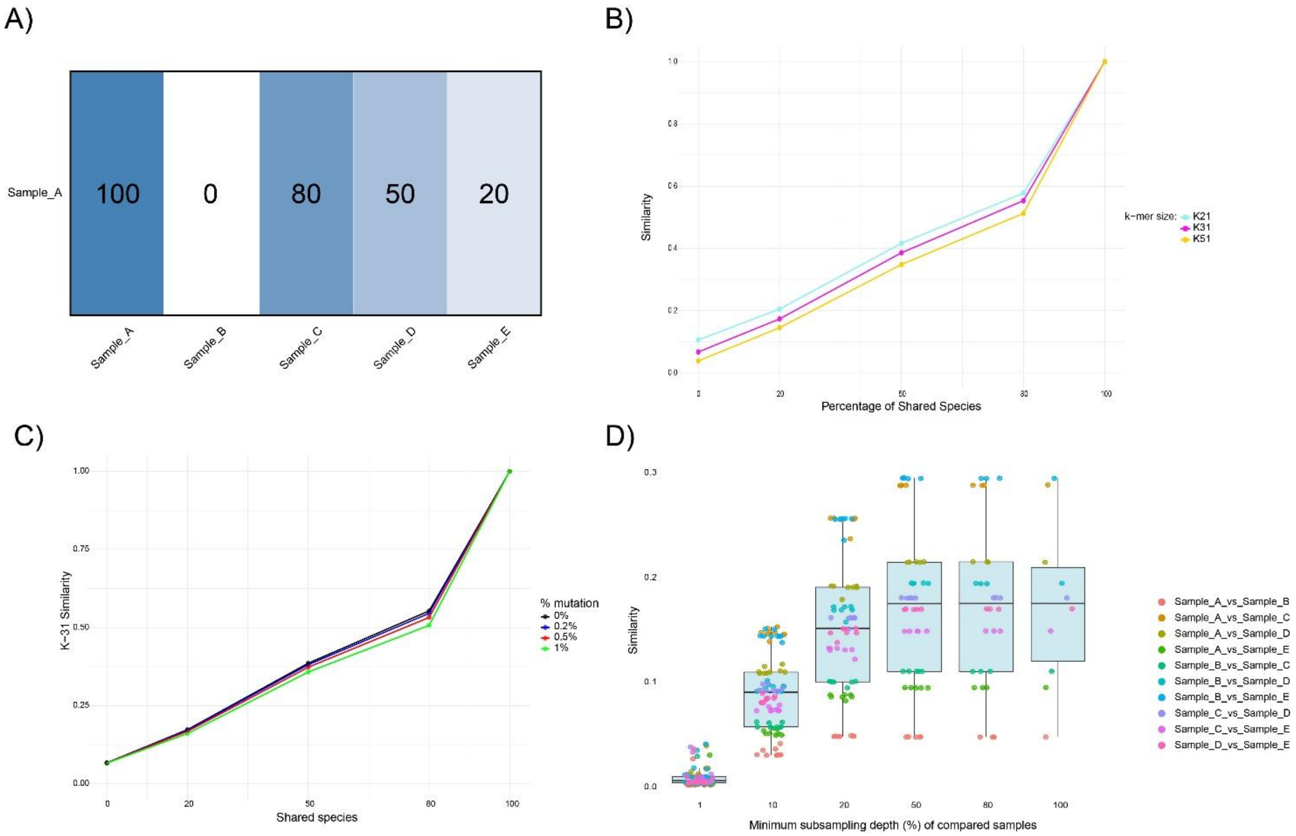

Vibrio K-mer Similarity Simulation using five synthetic metagenomes. Each synthetic metagenome consists of 30 *Vibrio* reference genomes from NCBI RefSeq. **A** Schematic representation of the percentage of shared genomes between Synthetic Metagenome A and the other synthetic metagenomes (B–E). Accession numbers and taxonomy of the *Vibrio* genomes used to generate synthetic metagenomes are reported in Table S11. **B** Different k-mer lengths (i.e., 21,31 and 51), **C** level of mutations and **D** subsampled samples were tested to provide cutoff for similarity values, which can help in the interpretation of the k-mer analysis applied in the study. For example, the results showed that similarity detected for the MiAV fraction (>0.4 at a travel time of 1.5 years) corresponded to >50% genome sharing between *Vibrio* communities, while for the FLV fraction (similarity <0.15 at a travel time of 1.5 years), this value dropped to <15% (see also Fig 3C). In **D**, Boxplot bounds indicate the interquartile range (IQR) defined as the difference between the first quartile (Q1) and the third (Q3), whiskers extend to the minimum and maximum values within [Q1−1.5×IQR, Q3+1.5×IQR], horizontal lines represent median values. Sample size: For 1 n= 110, for 10 n= 90, for 20 n= 70, for 50 n= 50, for 80 n= 30, for 100 n= 10.

Fig. S5.

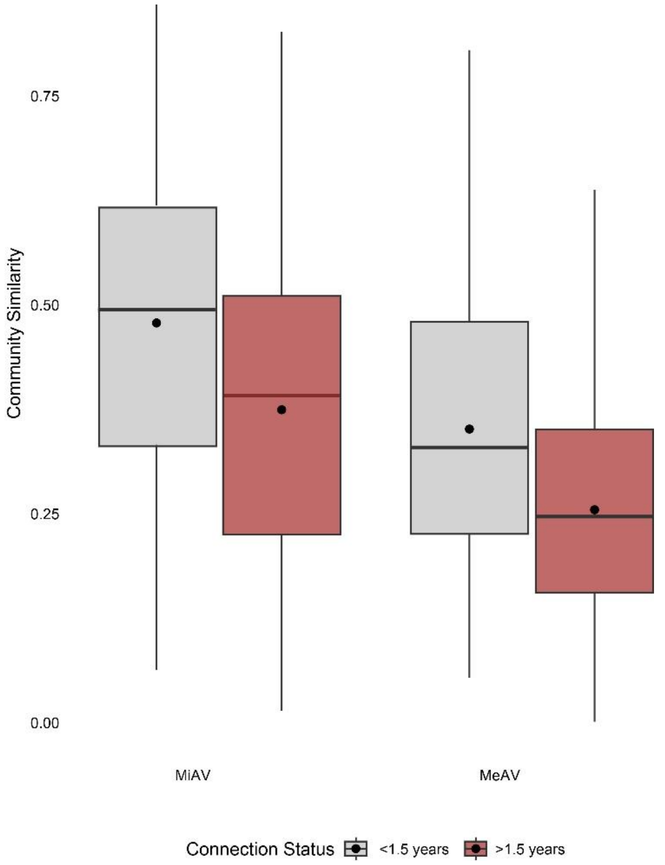

Comparison of Vibrio community similarity in MiAV (20–180 μm) and MeAV (180–2000 μm) fractions across travel time (TT) thresholds (1.5 years). Boxplots show *Vibrio* similarity values grouped by connection status (TT < 1.5 years vs TT ≥ 1.5 years). Boxplot bounds indicate the interquartile range (IQR) defined as the difference between the first quartile (Q1) and the third (Q3), whiskers extend to the minimum and maximum values within [Q1−1.5×IQR, Q3+1.5×IQR], horizontal lines represent median values and the black dot the mean. Sample sizes: n = 1319 (TT < 1.5 years) and n = 3674 (TT ≥ 1.5 years) for MiAV, and n = 1360 and n = 4286 for MeAV, respectively. Statistical differences between groups were assessed using two-sided Wilcoxon rank-sum tests. Both fractions showed significantly higher similarity values for TT < 1.5 years compared to TT ≥ 1.5 years (MiAV: W = 3003712, $p = 3.01 \times 10^{-38}$; MeAV: W = 3374894, $p = 1.48 \times 10^{-18}$).

Fig. S6.

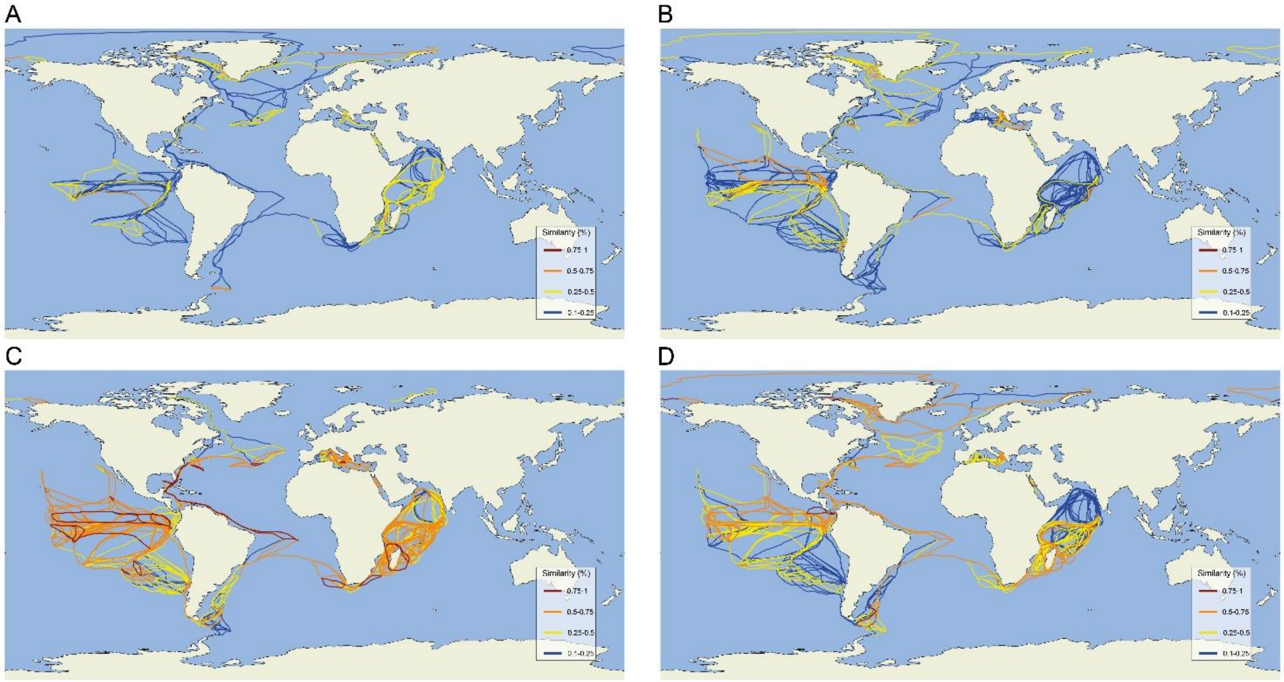

Networks of the Long-range Dispersals of *Vibrio* in the Oceans. Networks among stations connected by a travel time less than 1.5 years for the **A** FLV-0.22–3 µm, **B** NAV-5-20 µm, **C** MiAV-20-180 µm and **D** MeAV-180-2000 µm size fractions. Connections are color-coded based on the similarity of the *Vibrio* communities between stations.

Fig. S7.

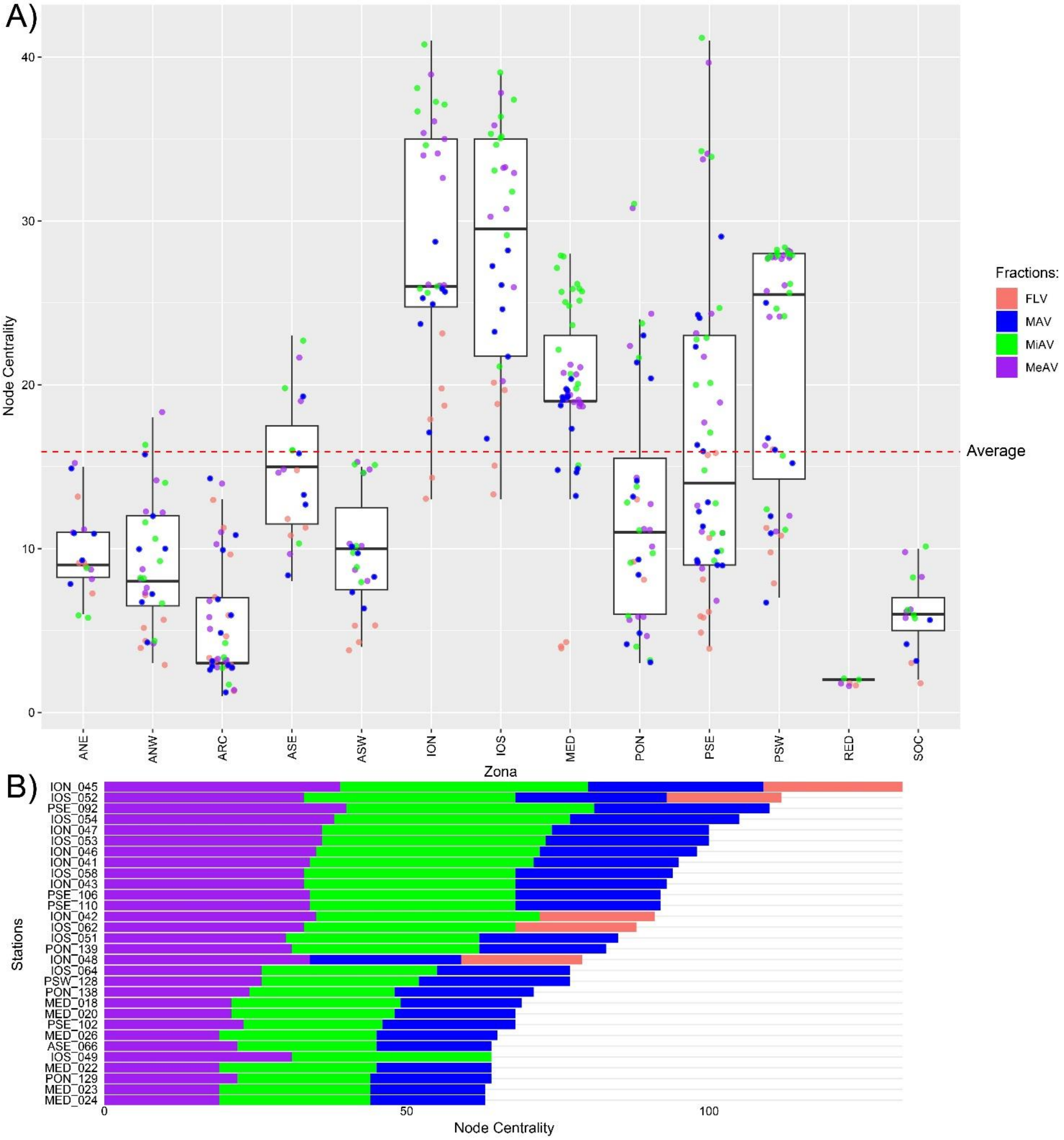

Degree Node Centrality Values for *Vibrio* Networks. **A** Distribution of node centrality degree for the superficial stations in each oceanic region The average node centrality degree (15.9038) is represented by the red dashed line. Boxplot bounds indicate the interquartile range (IQR) defined as the difference between the first quartile (Q1) and the third (Q3), whiskers extend to the minimum and maximum values within [Q1−1.5×IQR, Q3+1.5×IQR], horizontal lines represent median values. Sample sizes for each region are: ANE n = 18, ANW n = 31, ARC n = 40, ASE n = 19, ASW n = 23, ION n = 32, IOS n = 32, MED n = 47, PON n = 36, PSE n = 52, PSW n = 44, RED n = 6, and SOC n = 15. **B** Top 30 stations with higher values of node centrality. Colors reflect the fractions.

Fig. S8.

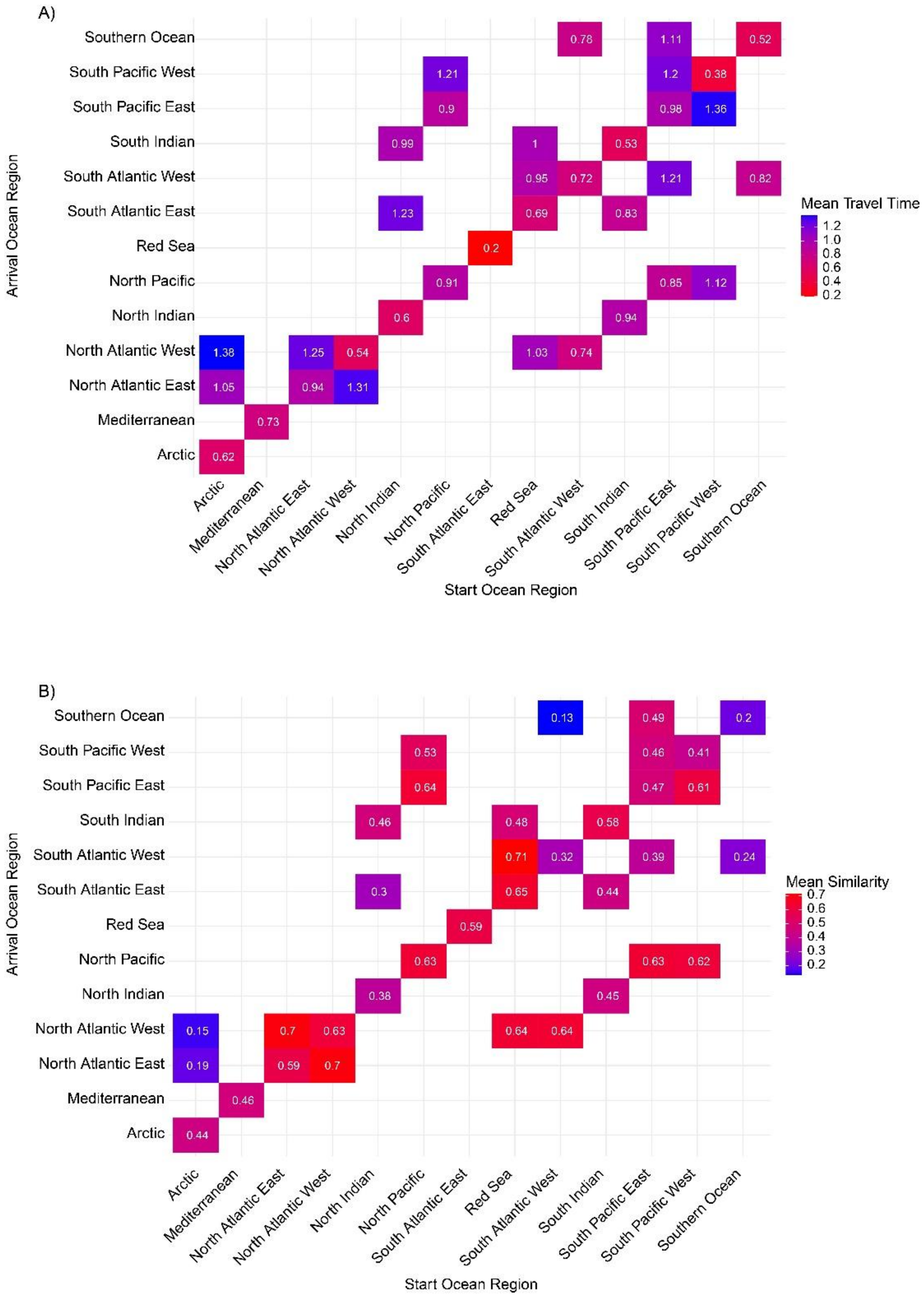

Travel time and Similarities Values. **A** Average travel time and **B** similarities for the MiAV (20-180 µm) fraction among sub oceanic regions.

Fig. S9.

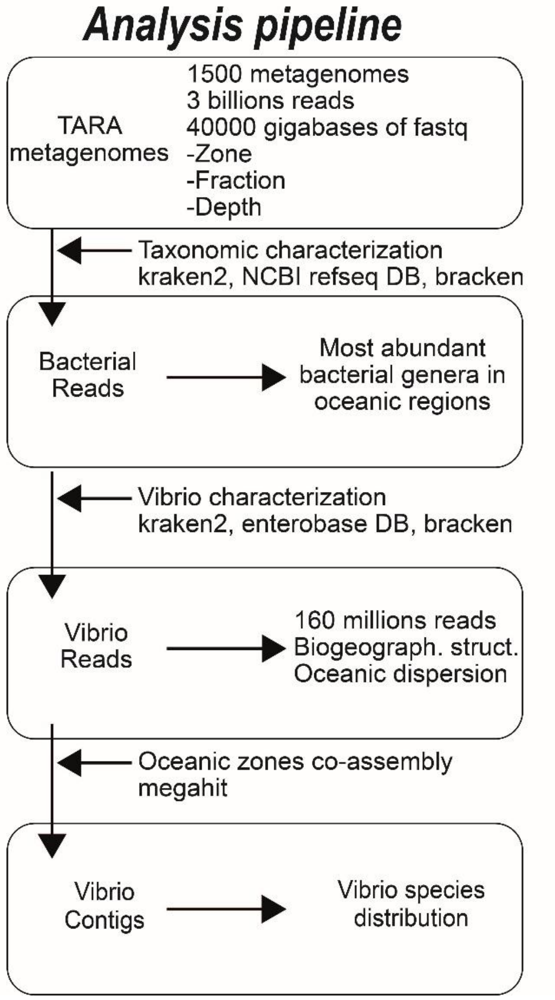

Brief Overview of the Pipeline Used to Analyze the TARA Oceans Metagenomes. For the detailed bioinformatic methods see the repository https://github.com/LDoni/Deciphering-the-Hidden-Ecology-of-Vibrio-in-the-Oceans.
