# Supplementary material for: Deciphering the Hidden Ecology and Connectivity of Vibrio in the Oceans
Source: Nat Commun. 2026 Apr 1;17:4707. doi: 10.1038/s41467-026-71231-3 (PMC13212558; doi:10.1038/s41467-026-71231-3)
Supplement: Supplementary file 2 — Descriptions of Additional Supplementary Files [file 41467_2026_71231_MOESM2_ESM.pdf]

Descriptions of Additional Supplementary Files

Supplementary Data S1: Metagenomic co-assemblies stats

Supplementary Data S2: Vibrio tpm frequency

Supplementary Data S3: Estimated travel times

Supplementary Data S4: SRF FLV similarity

Supplementary Data S5: SRF NAV similarity

Supplementary Data S6: SRF MiAV similarity

Supplementary Data S7: SRF MeAV similarity

Supplementary Data S8: Two-sided Fisher z-test similarity vs travel time

Supplementary Data S9: Two-sided Fisher z-test similarity vs Km

Supplementary Data S10: Linear regression statistics for z-scored TT and distance

Supplementary Data S11: MiAV long-distance biological corridors

Supplementary Data S12: Vibrio species used for the k-mers similarity simulations

Supplementary Movie 1: Long-range Dispersals of *Vibrio* in the surface Oceans. Video of the networks among stations connected by a travel time less than 1.5 years for the FLV-0.22–3 µm (A), NAV-5-20 µm (B), MiAV-20-180 µm (C) and MeAV-180-2000 µm (D) size fractions. Connections are color-coded based on the similarity of the *Vibrio* communities between stations.
