# Supplementary material for: Deciphering the Hidden Ecology and Connectivity of Vibrio in the Oceans
Source: Nat Commun. 2026 Apr 1;17:4707. doi: 10.1038/s41467-026-71231-3 (PMC13212558; doi:10.1038/s41467-026-71231-3)
Supplement: Supplementary file 5 — Reporting Summary [file 41467_2026_71231_MOESM5_ESM.pdf]

nature portfolio

Corresponding author(s): Luigi Vezzulli and Jaime Martinez-Urtaza

Last updated by author(s): Feb 17, 2026

Reporting Summary

Nature Portfolio wishes to improve the reproducibility of the work that we publish. This form provides structure for consistency and transparency in reporting. For further information on Nature Portfolio policies, see our Editorial Policies and the Editorial Policy Checklist.

Statistics

For all statistical analyses, confirm that the following items are present in the figure legend, table legend, main text, or Methods section.

n/a	Confirmed	
☐	☒	The exact sample size (*n*) for each experimental group/condition, given as a discrete number and unit of measurement
☐	☒	A statement on whether measurements were taken from distinct samples or whether the same sample was measured repeatedly
☐	☒	The statistical test(s) used AND whether they are one- or two-sided<br>*Only common tests should be described solely by name; describe more complex techniques in the Methods section.*
☐	☒	A description of all covariates tested
☐	☒	A description of any assumptions or corrections, such as tests of normality and adjustment for multiple comparisons
☐	☒	A full description of the statistical parameters including central tendency (e.g. means) or other basic estimates (e.g. regression coefficient) AND variation (e.g. standard deviation) or associated estimates of uncertainty (e.g. confidence intervals)
☐	☒	For null hypothesis testing, the test statistic (e.g. *F*, *t*, *r*) with confidence intervals, effect sizes, degrees of freedom and *P* value noted<br>*Give P values as exact values whenever suitable.*
☒	☐	For Bayesian analysis, information on the choice of priors and Markov chain Monte Carlo settings
☒	☐	For hierarchical and complex designs, identification of the appropriate level for tests and full reporting of outcomes
☐	☒	Estimates of effect sizes (e.g. Cohen's *d*, Pearson's *r*), indicating how they were calculated

Our web collection on statistics for biologists contains articles on many of the points above.

Software and code

Policy information about availability of computer code

Data collection	Metagenomic raw reads were downloaded from NCBI projects (PRJEB1787, PRJEB4352, PRJEB9740 582 and PRJEB9691), using bash utilities

Data analysis

In this study, all available shotgun metagenomes (about 1500) belonging to 4 NCBI TARA Oceans project (PRJEB1787, PRJEB4352, PRJEB9740 and PRJEB9691) were downloaded and analyzed, along with environmental metadata retrieved from the PANGAEA dataset (https://doi.pangaea.de/10.1594/PANGAEA.875579). Metagenomic reads were quality checked with trimgalore v0.6.7. The analysis of the bacterial composition was conducted with Kraken2 v2.1.2 using the Refseq NCBI Bacteria database (downloaded in 2021), and the frequencies were normalized using Bracken v2.6.2. Metagenomic reads classified as Vibrio were extracted using the extract_kraken_reads.py tool from KrakenTools v1.2. These reads were subsequently reclassified using a Kraken2 database, generated with the Vibrio genomes present in "The Vibrio database" from Enterobase in 2021, following the Kraken 2 manual. The frequencies of Vibrio species were normalized using Bracken. Data analyses and visualizations were performed primarily in R v4.3.3, using ggplot2 v3.5.2 for graphical representations, phyloseq v1.46.0 for diversity metrics, beta-diversity calculations including Bray-Curtis dissimilarities, PCoA, vegan v2.6-4 for PERMANOVA (adonis2), Mantel and Partial Mantel tests, and nlcor v.2.3 for computing the NonLinear Correlation Coefficient. Pairwise correlation differences between size fractions were evaluated using the diffcor v0.8.3 package via Fisher Z-tests. Additional visualizations and statistical summaries were generated with ggpubr v0.6.0, and igraph v1.5.1 was employed to analyze network centrality degree. Simka v1.5.3 was used to compute 31-mers (dis-)similarity across all Vibrio metagenomes. Travel Time among Tara stations was calculated using the Global Drifter Program interpolated dataset. Geographic distances between stations were calculated using the searoute.py v1.4.3. Oceanographic visualizations were generated using the Cartopy Python library v0.22.0. Co-assemblies were performed with MEGAHIT v1.2.9, and contigs were taxonomically classified using CAT (Contig Annotation Tool) v5.2.3. Contig abundances were quantified across all metagenomes using Salmon v1.9.0. The resulting abundance matrices were normalized using the relative log expression (RLE) method implemented in the MicrobiomeMarker R package v1.8.0. Statistical comparisons between groups were carried out using Wilcoxon and Kruskal-Wallis tests from the stats R package v4.3.3. https://github.com/LDoni/Deciphering-the-Hidden-Ecology-of-Vibrio-in-the-Oceans Zenodo repository: 10.5281/zenodo.14677762

For manuscripts utilizing custom algorithms or software that are central to the research but not yet described in published literature, software must be made available to editors and reviewers. We strongly encourage code deposition in a community repository (e.g. GitHub). See the Nature Portfolio guidelines for submitting code & software for further information.

Data

Policy information about availability of data

All manuscripts must include a data availability statement. This statement should provide the following information, where applicable:

- Accession codes, unique identifiers, or web links for publicly available datasets
- A description of any restrictions on data availability
- For clinical datasets or third party data, please ensure that the statement adheres to our policy

The metagenomic data used in this study are available in the NCBI TARA Oceans projects database under accession code PRJEB1787[www.ncbi.nlm.nih.gov/bioproject/196960], PRJEB4352[https://www.ncbi.nlm.nih.gov/bioproject/213098], PRJEB9740[https://www.ncbi.nlm.nih.gov/bioproject/288558] and PRJEB9691[https://www.ncbi.nlm.nih.gov/bioproject/287904].

Research involving human participants, their data, or biological material

Policy information about studies with human participants or human data. See also policy information about sex, gender (identity/presentation), and sexual orientation and race, ethnicity and racism.

Reporting on sex and gender: NA

Reporting on race, ethnicity, or other socially relevant groupings: NA

Population characteristics: NA

Recruitment: NA

Ethics oversight: NA

Note that full information on the approval of the study protocol must also be provided in the manuscript.

Field-specific reporting

Please select the one below that is the best fit for your research. If you are not sure, read the appropriate sections before making your selection.

☐ Life sciences ☐ Behavioural & social sciences ☒ Ecological, evolutionary & environmental sciences

For a reference copy of the document with all sections, see nature.com/documents/nr-reporting-summary-flat.pdf

Ecological, evolutionary & environmental sciences study design

All studies must disclose on these points even when the disclosure is negative.

Study description

This study aimed to uncover the global long-range dispersal patterns of the Vibrio genus in the oceans. Vibrio genus includes some of the most threatening human and animal pathogens of marine origin, making its spatial distribution and connectivity of particular

	concern. By combining 40 terabases of metagenomic data with satellite-tracked surface drifter data collected worldwide, the study revealed that Vibrio are abundant members of the ocean surface microbiome and show a strong association with microplankton, which likely governs their distribution and connectivity at the global scale. The analysis identified long-distance biological corridors linking Vibrio communities, including potentially pathogenic Vibrio, across thousands of kilometers in a relatively short time, estimated of less than 1.5 years to cross an ocean basin. These findings have deep implications for the demography and community dynamics of Vibrio species and the epidemiology of associated diseases.
Research sample	Metagenomes from the TARA Oceans expedition were used to analyze the Vibrio communities as describe in the metodology.
Sampling strategy	The general aim of the TARA Oceans expedition was to assess the complexity of ocean life across comprehensive taxonomic and spatial scales, sampling the oceans world-wide following standardized protocols for the collection and for data production. The methodology used for the sampling, size fractionation, DNA extraction and shotgun metagenomic sequencing has been already extensively described (Pesant et al., Sci. Data 2015; Sunagawa et al., Science 2015; Sunagawa, et al., Nat. Rev. Microbiol. 2020). For the metagenomic data used in this analysis (1500 metagenomes corresponding to 40Tbases of FASTQ, samples from each station were generally collected at three different depths (surface, deep chlorophyll maximum and mesopelagic) and serially filtered to obtain a prokaryotic-enriched fraction (0.22–3 µm) and three eukaryotic-enriched fractions (5–20 µm, 20–180 µm, and 180–2000 µm). Sample sizes were determined by the expedition's experimental design, which aimed to maximize spatial coverage and capture global diversity in relevant oceanic areas.
Data collection	The data collection is brieftly explained in the methods and extensively in Pesant et al., 2015 doi: 10.1038/sdata.2015.23.
Timing and spatial scale	The Tara Oceans expedition was conducted from 2009 to 2013, covering approximately 140,000 kilometers across all the world's oceans.
Data exclusions	No data were excluded from the analysis
Reproducibility	All metagenomes used are availables at NCBI projects (PRJEB1787, PRJEB4352, PRJEB9740 582 and PRJEB9691.), the methods to reproduce the manuscript's results are described in detail in the manuscript, and the code used is available at: https://github.com/LDoni/Deciphering-the-Hidden-Ecology-of-Vibrio-in-the-Oceans and in the Zenodo repository: 10.5281/zenodo.14677762.
Randomization	Does not apply
Blinding	Does not apply

Did the study involve field work? ☐ Yes ☒ No

Reporting for specific materials, systems and methods

We require information from authors about some types of materials, experimental systems and methods used in many studies. Here, indicate whether each material, system or method listed is relevant to your study. If you are not sure if a list item applies to your research, read the appropriate section before selecting a response.

Materials & experimental systems

n/a	Involved in the study	
☒	☐	Antibodies
☒	☐	Eukaryotic cell lines
☒	☐	Palaeontology and archaeology
☒	☐	Animals and other organisms
☒	☐	Clinical data
☒	☐	Dual use research of concern
☒	☐	Plants

Methods

n/a	Involved in the study	
☒	☐	ChIP-seq
☒	☐	Flow cytometry
☒	☐	MRI-based neuroimaging

Plants

Seed stocks	NA
Novel plant genotypes	NA
Authentication	NA
